# Editorial: Comorbidities and Aortic Valve Stenosis: Molecular Mechanism, Risk Factors and Novel Therapeutic Options

**DOI:** 10.3389/fcvm.2021.811310

**Published:** 2022-01-04

**Authors:** Cynthia St. Hilaire, Felix Jansen, Claudia Goettsch

**Affiliations:** ^1^Division of Cardiology, Departments of Medicine and Bioengineering, Vascular Medicine Institute, University of Pittsburgh, Pittsburgh, PA, United States; ^2^Heart Center Bonn, Department of Medicine II, University Hospital Bonn, Bonn, Germany; ^3^Department of Internal Medicine I—Cardiology, Medical Faculty, RWTH Aachen University, Aachen, Germany

**Keywords:** aortic valve stenosis, comorbidities, fibrosis, calcification, risk factors, clinical challenges, therapy

## Introduction

Aortic valve stenosis (AS) caused by calcific aortic valve disease (CAVD) is the most prevalent heart valve disease of the elderly population in the Western world, and the disease burden is estimated to increase from 2.5 million in 2000 to 4.5 million in 2030 (1). Fibro-calcific remodeling is a hallmark of disease progression and a strong independent prognostic marker for adverse events in patients with asymptomatic AS. Currently, no medication exists to halt or even slow AS and CAVD progression. The only therapeutic options available are invasive—either surgical or catheter-based aortic valve replacement—thus, the CAVD research field focuses on discovering novel CAVD biomarkers and targets for developing non-invasive therapies [reviewed in (2)].

## Novel Mechanism of Calcific Aortic Valve Disease

CAVD was once considered a passive degenerative process resulting from chronic mechanical stress. However, several decades of research now show that CAVD is an active, highly regulated, and progressive process characterized by a cascade of cellular changes that initially cause fibrotic thickening, followed by extensive calcification of the aortic valve leaflets. Büttner et al. provide an updated review on the cellular and molecular drivers of early fibrogenesis in CAVD, focusing on the role of biomechanical forces, cellular contribution, risk factors, and sex differences driving pathogenic remodeling, as well as imaging valvular fibrosis.

Two publications report on novel mechanisms driving CAVD. Roos et al. assessed the role of manganese superoxide dismutase (MnSOD) as a source of mitochondrial-derived oxidative stress in the AS progression in the *Ldlr*-deficient/*ApoB*^100/100^ mice model. MnSOD inactivation or overexpression did not affect valve and ventricular function *per se*. Interestingly, reducing MnSOD accelerated valvular calcification while overexpression of MnSOD did not provide any protective effects. The complex nature of these findings indicated that the balance of mitochondrial oxidative stress is essential for maintaining valvular homeostasis. This study also underlines the shared mechanism of osteogenesis and ectopic calcification since it was shown that MnSOD is required to regulate mitochondrial stress during bone formation (3).

Dharmarjan et al. assessed the role of *RUNX2*, a well-known osteogenic transcription factor, in the *Ldlr*-deficient/*ApoB*^100/100^ mice and demonstrated that in valve interstitial cells (VICs), SM22 is a marker for “activated” VICs, and SM22+ cells go on to express osteochondrogenic genes in the valve. Depleting *RUNX2* in SM22+ cells reduced calcification in the leaflet hinge and sinus wall and improved hemodynamic parameters; however, the valve cusp in *Ldlr*-deficient/*ApoB*^100/100^ mice did not exhibit significant calcification. Thus, this study importantly identifies that leaflet hinge and sinus wall calcification is sufficient to alter valve dysfunction.

Various cellular and molecular mechanism are described in CAVD that might be all interlinked. For example, it was shown that redox pathways regulate RUNX2 transcription factor activity in endothelial cells (4). Future efforts may consider studying the connection between established molecular pathways involved in CAVD. This approach might identify novel center players with therapeutic potential.

## Diagnostic Markers of Aortic Stenosis

In daily clinical practice, echocardiographic, and laboratory parameters have been shown to have some prognostic value in severe AS. However, these are utilized only when AS symptoms present themselves, and there is still an unmet need for robust prognostic biomarkers or predictors for identifying asymptomatic AS patients with poor prognoses. Chen et al. identified plasma tissue plasminogen activator (t-PA)—an indicator for impaired fibrinolysis, as a biomarker for AS. The abundance of t-PA was also higher in fibrotic or calcified aortic valve leaflets than non-diseased leaflets. This finding aligns with a previous report demonstrating an association between high t-PA levels and AS (5).

Mas-Peiro et al. assessed the prognostic value of baseline endogenous erythropoietin (EPO) for the survival of patients with severe AS undergoing transcatheter aortic valve replacement (TAVR). High baseline EPO levels predict mid-term mortality in patients undergoing TAVR independent of anemia and moderate kidney impairment. Studies with a larger sample size are needed to examine whether t-PA and EPO could serve as robust clinical diagnostic AS markers. Whether t-PA and EPO are also actively involved in the pathophysiological mechanisms of AS is unknown.

## Clinical Decision-Making to Manage Patients With Aortic Stenosis

Yuan et al. performed a meta-analysis including 1,249 participants and found a benefit of an early interventional strategy for asymptomatic AS patients with preserved left ventricular ejection fraction. Wu et al. found that in-hospital mortality and stroke were similar with TAVR and surgical aortic valve replacement in AS patients with arterial fibrillation.

Mauri et al. reported an incidence of 10% for patients with post-operative delirium (POD) after TAVR that adversely impacted the 2-year survival. Male sex, atrial fibrillation frailty, stroke, pneumonia, and vascular complications were identified as the risk factors of POD. Taken together, co-morbidities, age, and frailty contribute to the clinical decision-making of aortic valve replacement time and procedure. There is an improved outcome with earlier intervention.

## Conclusions

The contributions in this Research Topic illustrate the complexity of the mechanism and clinical management of AS. Critically important is further defining the initial drivers of osteogenic switching of valvular cells, as well as the propagation of calcification throughout the leaflet. Increasing evidence suggest sex-specific influences underly some of the cellular and molecular mechanism in AS and CAVD (Büttner et al.), however the means by which sex hormones contribute to the initiation or progression of CAVD are unknown. To address these questions it is imperative that we identify and develop more representative *in vivo* or *ex vivo* models to study this process. The findings by Dharmarjan et al. clearly show a role for RUNX2 activity and the myofibroblastic transition of valve interstitial cells, however, the localization and patterning of calcification in the murine aortic valve is distinct from human CAVD pathology. This could perhaps be due to the background—the *Ldlr*-deficient/*ApoB*^100/100^ mice is an atherosclerotic model—or to perhaps innate differences between mice and humans (6).

Furthermore, the notion that early intervention improves clinical outcomes underlines the need for imaging modalities to diagnose early-stage aortic valve remodeling in asymptomatic patients (Büttner et al.). Focusing on the development of molecular probes and high-resolution imaging to detect early tissue changes, such as fibrotic remodeling, are necessary for the development of screening tools.

Given the increasing number of patients with these complex disease states, there is also an urgent need to better understand how complex co-morbidities influence the mechanism of AS initiation and progression of CAVD pathogenesis. Hypertension, type 2 diabetes mellitus, dyslipidemia, chronic kidney disease, chronic inflammatory diseases, and anemia are co-morbidities highly prevalent in AS patients (7–10). The study by Roos et al. identified a role for mitochondrial-derived oxidative stress in CAVD progression, suggesting a potential link between calcification processes related to metabolic shifts seen in obese or diabetic patients. A focus on interorgan crosstalk, or the development of organoid or *ex vivo* models may also help to expand our understanding of how disease states influence CAVD pathogenesis. With a greater understanding of the steps that a cell takes in its transition from a healthy to calcified state we will be able to leverage these new discoveries for the development of novel therapeutic options to treat CAVD and AS.

## Author Contributions

All authors contributed to the editorial and approved the submitted version.

## Funding

This work was funded by National Institutes of Health R01 HL142932 to CS, American Heart Association 20IPA35260111 to CS, Deutsche Forschungsgemeinschaft (JA 2351/2-1, Project-ID 397484323-TRR 259 to FJ and GO1801/5-1, Project-ID 322900939-TRR219 to CG), and Corona-Foundation to FJ.

## Conflict of Interest

The authors declare that the research was conducted in the absence of any commercial or financial relationships that could be construed as a potential conflict of interest.

## Publisher's Note

All claims expressed in this article are solely those of the authors and do not necessarily represent those of their affiliated organizations, or those of the publisher, the editors and the reviewers. Any product that may be evaluated in this article, or claim that may be made by its manufacturer, is not guaranteed or endorsed by the publisher.
